# Ecological stoichiometry of the honeybee: Pollen diversity and adequate species composition are needed to mitigate limitations imposed on the growth and development of bees by pollen quality

**DOI:** 10.1371/journal.pone.0183236

**Published:** 2017-08-22

**Authors:** Michał Filipiak, Karolina Kuszewska, Michel Asselman, Bożena Denisow, Ernest Stawiarz, Michał Woyciechowski, January Weiner

**Affiliations:** 1 Institute of Environmental Sciences, Jagiellonian University, Kraków, Poland; 2 Department of Botany, Laboratory of Horticultural Plant Biology, University of Life Sciences in Lublin, Lublin, Poland; International Nutrition Inc, UNITED STATES

## Abstract

The least understood aspects of the nutritional needs of bees are the elemental composition of pollen and the bees’ need for a stoichiometrically balanced diet containing the required proportions of nutrients. Reduced plant diversity has been proposed as an indirect factor responsible for the pollinator crisis. We suggest stoichiometric mismatch resulting from a nutritionally unbalanced diet as a potential direct factor. The concentrations and stoichiometric ratios of C, N, S, P, K, Na, Ca, Mg, Fe, Zn, Mn, and Cu were studied in the bodies of honeybees of various castes and sexes and in the nectar and pollen of various plant species. A literature review of the elemental composition of pollen was performed. We identified possible co-limitations of bee growth and development resulting mainly from the scarcity of Na, S, Cu, P and K, and possibly Zn and N, in pollen. Particular castes and sexes face specific limitations. Concentrations of potentially limiting elements in pollen revealed high taxonomic diversity. High floral diversity may be necessary to maintain populations of pollen eaters. Single-species crop plantations, even if these species are rich in nectar and pollen, might limit bee growth and development, not allowing for gathering nutrients in adequate proportions. However, particular plant species may play greater roles than others in balancing honeybee diets. Therefore, we suggest specific plant species that may (1) ensure optimal growth and production of individuals by producing pollen that is exceptionally well balanced stoichiometrically (e.g., clover) or (2) prevent growth and development of honeybees by producing pollen that is extremely unbalanced for bees (e.g., sunflower). Since pollen is generally poor in Na, this element must be supplemented using “dirty water”. Nectar cannot supplement the diet with limiting elements. Stoichiometric mismatch should be considered in intervention strategies aimed at improving the nutritional base for bees.

## Introduction

The growth and development of any organism may be limited by unbalanced stoichiometry, namely unbalanced proportions of atoms of chemical elements in food that reflect proportions of physiologically important organic molecules [1–4]. According to the law of conservation of mass, specific atoms (in contrast to organic compounds) are not converted to other elements during processing of the consumed matter. Thus, a stoichiometric mismatch can occur between the elemental composition of the consumer’s body and that of its food, thereby limiting the consumer’s growth and development [2–5]. Observed “toxic” effects on an organism by a particular diet may in fact be caused by stoichiometric mismatch, i.e., low nutritional value of the diet, rather than by toxic substances [6]. Hence, maintaining the balance between matter supply and demand is crucial for the development of an organism and, therefore, building the body of its adult form. In this context, herbivores must cope with stoichiometric mismatches resulting from the fundamental differences between the elements ratios of their tissues and food [1,2,7] (see [5,8,9] for comparison with other feeding guilds). Such incompatibility may result in hampered growth rates and decreased survivorship and reproduction, thereby influencing the fitness of the consumer. However, the least understood aspect of the nutritional needs of bees concerns stoichiometric balancing and the need for adequate amounts and ratios of particular nutritional elements in consumed food [10,11] c.f. [1–4,6,7,12]. The vast majority of the non-carbon elements used to build the tissues of growing bee larvae originate from pollen [13–15]. Nurse bees (workers that feed larvae) ingest pollen (fresh or stored as beebread), nectar and water. Nectar serves as the source of energy, whereas pollen is the source of nutrients required to build and maintain the bodies of the bees [13–15]. Therefore, considering the framework of ecological stoichiometry, nectar is the source of C, H and O, and pollen is the source of other elements composing organic molecules. Digested compounds are used to make jelly, i.e., the food of honeybee larvae, which is excreted by nurse bees. The jelly constitutes the exclusive food of one- to three-day-old larvae and the majority of food (mixed with honey and pollen) of older larvae [16]. Considering the possible sources of chemical elements gathered by bees (nectar, pollen, water), it can be assumed that the stoichiometry of pollen is the key factor influencing the nutritional balance of larval food, since (1) energy is readily available from nectar; (2) pollen is almost the exclusive source of non-carbon elements for bees; (3) elements are conserved, i.e., they cannot be converted into other elements; and (4) organic compounds are processed and recycled by jelly-producing nurse bees. During the larval growth period, all chemical elements that form the body of the adult must be assimilated. This process requires the appropriate proportions of elements to be provided in the food. Thus, the development of bee larvae may be limited by the amount and stoichiometry [2,3] of the food provided by nurse bees, and the original source of this limitation is the elemental composition of the pollen from which this food was made. For organisms that feed on plant matter, the quality (elemental ratios) may be more limiting than the quantity of the plant matter [2,9,17]; in other words, the growth and development of these organisms may not be limited primarily by energy but instead by the proportion of the body-building nutrients in food.

Nutritional stress has been suggested to be responsible for honeybee colony collapse [13,18]. Such stress may be associated with the non-random selection of species of nutritional plants by bees [19]. Recently, the differences in the nutritional quality of various pollen species for Apidae were studied (e.g., [20–22]) but without regard to the stoichiometric relations between pollen and pollen eaters. Studies of the elemental composition of commercial pollen, a human dietary supplement, have shown a high taxonomic variability of pollen stoichiometry (e.g., [23–25]). This variability may have negative consequences for bees: a specific pollen may be not balanced stoichiometrically, i.e., may be deficient in necessary nutritional elements while having a surplus of others. However, a diet composed of various proportions of different pollen species might allow bees to achieve a stoichiometrically balanced element budget. Therefore, the reduction of plant diversity may be an important factor driving the decline of bees by imposing stoichiometric limitations on bee development. These limitations may be different for different castes. Honeybee individuals develop along one of three possible life histories (drones—males, workers–infertile females, and queens–fertile females [26–28]; hereafter called castes and sexes). The different biological functions of these castes and sexes require specific allocations of elements, which should be reflected by elemental ratios. Thus, the different life history traits of honeybee individuals should be reflected in differences in body stoichiometries, and available matter should be invested differentially among the bodies of growing larvae.

To fully explain how pollen stoichiometry may affect larval development, direct experimental measurements of element balances are needed. Such measurements require large numbers of experiments, utilizing various diets that are poor or enriched in atoms of particular elements, multiplied by the number of elements studied. Such data do not currently exist for the honeybee or for invertebrates in general. However, to detect potential stoichiometric mismatches and their consequences for larvae of various castes and sexes, simple comparisons of element ratios are sufficient, utilizing the *TSR* index, which indicates a possible limitation imposed on the growth and development of an organism that feeds on a given food [29].

The aims of this study were to (1) investigate variability in the concentrations of 12 elements (C, N, S, P, K, Na, Ca, Mg, Fe, Zn, Mn, and Cu) in pollen, (2) identify those nutritional elements that may limit bee development, and (3) quantitatively assess the relationship between the stoichiometries of particular life strategies of bee castes and sexes with the stoichiometries of the pollen of diverse plant species. We hypothesize that (1) the pollen stoichiometry of single species imposes a limiting stoichiometric mismatch on bees, with differing mismatches among various taxa of pollen; (2) different castes and sexes of honeybee experience different mismatches; and (3) polyfloral pollen is needed to build the honeybee imago because such pollen allows stoichiometric balance. The study consisted of two research tasks. Task (1) was planned as a field experiment in which we measured the concentrations of 12 elements in the bodies of 3 castes and sexes of honeybees (queens, drones and workers), in the nectar consumed by bees, and in the pollen pellets (pollen loads) collected by bees, which were composed of various species of pollen. Task (2) was based on data in the literature. This approach allowed the study of elemental composition and stoichiometry of various species of pollen that were collected worldwide (data collection of a scope that would be impossible within a single study). We collected published data on the elemental composition of the pollen of various plant species. Based on these data, we tested whether pollen stoichiometry may limit the development of bees by calculating the stoichiometric mismatches imposed on the bees by taxonomically different pollen. If various pollen species are scarce in different elements, they should impose different limitations on bee development. If they are also rich in different elements, combinations of various pollen species should allow for a stoichiometrically balanced diet. Thus, appropriate compositions and diversities of flora may promote bee development regardless of the quantity of pollen and nectar produced. If so, intervention strategies aimed at providing nutritional support for bees should consider not only the quantity of pollen and nectar produced by plants but also the quality of pollen (which may be reflected as pollen stoichiometry).

## Materials and methods

### Study setup

The study consisted of two separate research tasks, (1) a field experiment investigating the possible limiting effects imposed on bee development by the stoichiometry of available and collected pollen and (2) a literature review investigating the variance in the stoichiometrically limiting effects of the pollen produced by different plant taxa.

The first task was performed to identify nutritional elements that, if scarce in pollen, may limit honeybee growth and development and to investigate if honeybees mix pollen with unfavorable stoichiometry from various species to obtain a favorable and stoichiometrically balanced polyfloral pollen blend. Additionally, the composition of nutritional non-carbon elements in collected nectar was studied to investigate whether nectar may be a source of non-C body-building elements for bees.

The second task was performed to investigate the taxonomical variability of pollen stoichiometry and to identify the plant species that produce either limiting or favorable pollen.

### Research task (1): Experiment

#### Study site and specimen collection

The field component of the research was performed from May to July 2014 in the experimental apiary of the Institute of Environmental Sciences (Jagiellonian University, Kraków, Poland; 50° 01' 35'' N; 19° 54' 05'' E; elevation 213 m.a.s.l., mean annual temperature: 8.7°C; mean annual precipitation: 679 mm). All of the chemical analyses of insect bodies used newly hatched, freeze-dried imagines of honeybees (*Apis mellifera carnica*; queens, workers and drones) that had not eaten after emergence; to ensure this, brood combs after pupa formation were carried to the lab and incubated at 32°C until emergence. These bees (205 specimens in total) were collected from three colonies. We also analyzed the pollen and nectar that were collected by bees at the study site during the period of bee development under study. Another bee colony was used as a source of the pollen pellets (i.e., pollen loads collected with pollen traps mounted at the entrance to the hive), and 5 additional bee colonies were used as the sources of nectar. This procedure ensured that the growth and development of the bees (sampled for chemical analysis as adults) were not disturbed. Pollen loads and nectar samples were collected every 3 days during the development of the bees (which were sampled for chemical analysis as adults). The fresh nectar (i.e., not concentrated into honey) could be only collected as the matter gathered and stored by the bees in the beehives. These colonies were not disturbed in any way other than through the collection of pollen and nectar. Additionally, we analyzed commercial pollen pellets (sold as a dietary supplement) that were collected from honeybees in Poland.

#### Pollen pellet morphospecies (*PPMs*)

**Fig 1:**
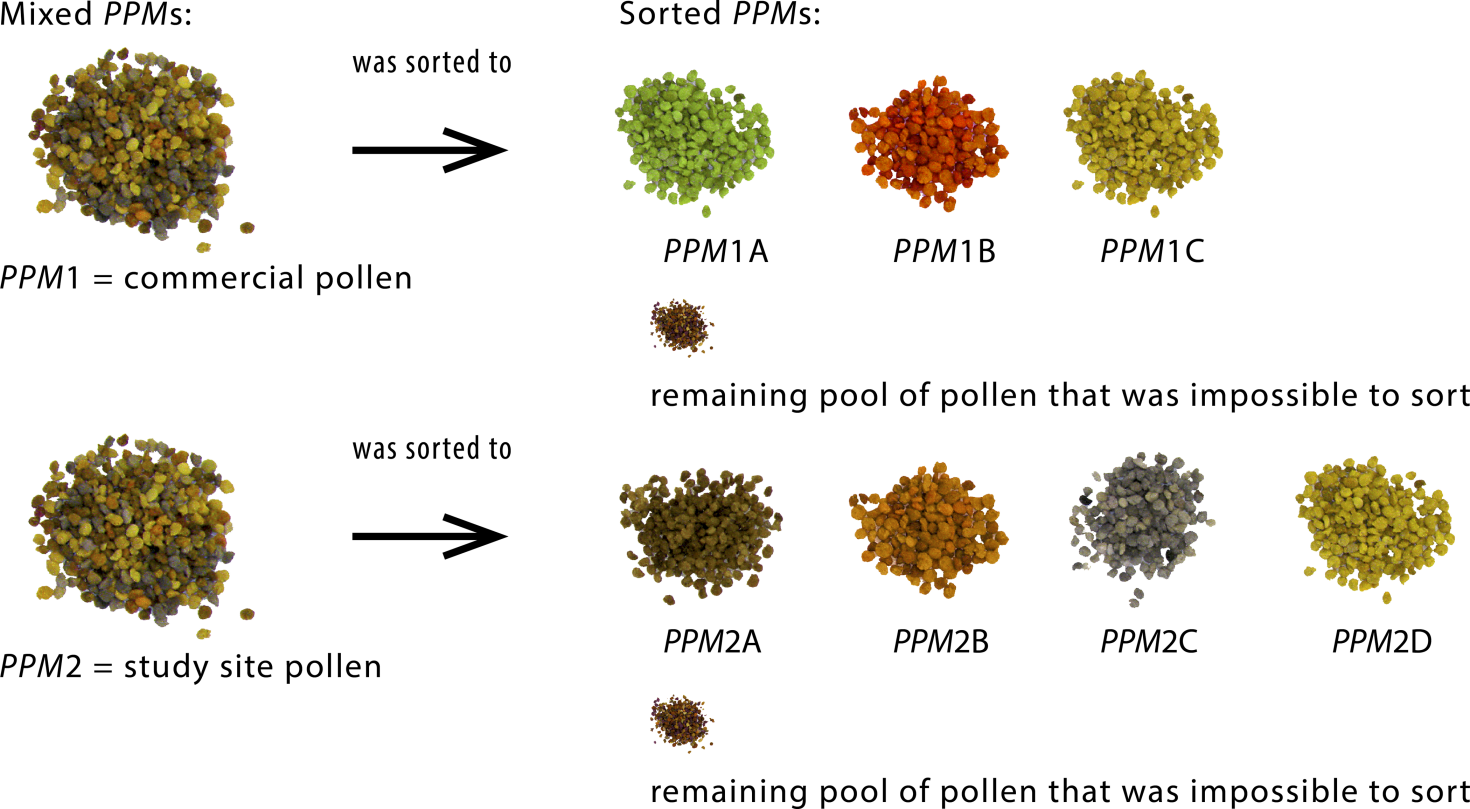
Symbolic representation of the process of pollen sorting from mixed to sorted *PPMs*.

The pollen samples belonged to 2 pools, (i) pollen that was collected by honeybees as pollen pellets in various apiaries and available commercially as a human dietary supplement (commercial pollen) and (ii) pollen that was collected by honeybees as pollen pellets at the study site (study site pollen) and represented the pollen available for the studied bee colonies (Fig 1). These two pools were called *PPMs*, in which *PPM1* = commercial pollen and *PPM2* = study site pollen. *PPM1* and *PPM2* were called mixed *PPMs* because they represented the most polyfloral mixes of pollen species. From both mixed *PPMs*, sorted *PPMs* were obtained and divided according to color by the naked eye as follows: *PPM1A*, green; *PPM1B*, red; *PPM1C*, yellow; *PPM2A*, bronze; *PPM2B*, orange; *PPM2C*, gray; and *PPM2D*, yellow. Thus, mixed *PPMs* 1 and 2 constituted the pool of pollen that was collected by the honeybees, and sorted *PPMs* had specific species compositions that differed from those of mixed *PPMs* but contributed to those of mixed *PPMs* (Fig 1). This approach allowed us to investigate whether honeybees might mix pollen with different stoichiometries in proportions that would produce a stoichiometrically balanced pollen mix. Using this procedure, every morphospecies sample could be obtained in sufficient masses for elemental measurements, without contamination from chemical reagents. The exact species composition (the percentage of every noted taxon) of each distinct *PPM* was estimated by counting the pollen grains under a microscope using samples of 3.5 g dry mass (d.m.) of each homogenized *PPM* following the recommendations of the International Commission for Bee Botany of IUSB [30]. The results are presented in S3 Table.

#### Chemical analysis

Bees and pollen samples were freeze-dried. We determined the C, N and S concentrations using a Vario EL III automatic CHNS analyzer. The K, Ca, Mg, Fe, Zn, Mn, Cu and Na concentrations were determined by atomic absorption spectrometry (Perkin-Elmer AAnalyst 200 and Perkin-Elmer AAnalyst 800), and the P content was determined by colorimetry (FIA: MLE FIA flow injection analyzer). For pollen, we used ground and homogenized pollen pellets, which allowed us to form samples of sufficient mass for analysis (approx. 8 mg d.m. for C, N and S analysis and approx. 1 g d.m. for the remaining elements; in total, 57 samples were used for C, N and S analysis, and 82 samples were used for the remaining elements). The bee samples consisted of one to five individuals (depending on the specimen sizes and measurement requirements). In total, 47 samples, each consisting of 1 individual, were used for C, N and S analysis, and 48 samples, each consisting of 1 to 5 individuals, were used for the analyses of concentrations of the remaining elements (this was done for practical reasons, i.e., (1) minimal and maximal concentrations of elements that can be measured utilizing different devices; (2) different procedures for sample preparation and processing for C, N and S analysis and for the analyses of the remaining elements). The supplemental materials indicate the number of samples measured for every element, pollen morphospecies and bee sex and caste (bees: S1 Table; pollen: S4 Table). To analyze 9 elements (P, K, Na, Ca, Mg, Fe, Zn, Mn and Cu) in liquid samples (suitable for AAS and FIA but not for CHNS), samples were mineralized by acid digestion with a 4:1 solution of nitric acid (70%) and hydrogen peroxide (30%), followed by hotplate digestion. Sulfanilic acid was used as the reference material for the C, N and S analyses, and certified reference materials (bush, NCS DC 73349; chicken, NCS ZC 73016; and bovine muscle powder, RM8415) were used for the other elements. From nectar, it was only possible to analyze 9 non-carbon elements (P, K, Na, Ca, Mg, Fe, Zn, Mn and Cu) in liquid samples (suitable for AAS and FIA, but not for CHNS; 24 samples were used, at 4 ml of nectar per sample).

#### Trophic stoichiometric ratio (*TSR*)—The index of stoichiometric mismatch

The degree of stoichiometric mismatch between the bees and pollen for each element *x* was expressed using the index of trophic stoichiometric ratio [29], *TSR*, a modified version of the threshold elemental ratio (*TER)*; according to previous studies [2,5,8,31]:

$$TER_{x}=\left(GGE_{x}/GGE_{C}\right)\times {\left(C:X\right)}_{i+1}$$ (1)

where *GGE*_*x*_ is the gross growth efficiency of the element *x*, *GGE*_*C*_ is the gross growth efficiency of carbon, *i* is the trophic level, *C* is the concentration of carbon and *X* is the concentration of element *x*.

If

$${\left(C:X\right)}_{i}\geq TER_{x}$$ (2)

then element *x* may become a limiting factor for growth at trophic level *i+1*. The gross growth efficiencies for any given element can be experimentally measured by feeding trials in growing animals. However, such data are extremely scarce for elements other than N and P. In practical terms, the *TER* index for invertebrates can only be estimated using arbitrary assumptions [8,32,33]. Therefore, we use *TSR*, a simpler index based on readily available data [29], to enable the detection of possibly limiting stoichiometric mismatches between trophic levels and comparisons between various taxa, habitats and foods. We propose the following simplified approach, rewriting condition (2)

$${\left(C:X\right)}_{i}\geq \left(GGE_{x}/GGE_{C}\right)\times {\left(C:X\right)}_{i+1}$$

to obtain

$${\left(C:X\right)}_{i}/{\left(C:X\right)}_{i+1}\geq GGE_{x}/GGE_{C}\;\mathrm{or}\;TSR_{x}\geq GGE_{x}/GGE_{C}$$

Even without precise information regarding the elemental conversion efficiencies for a given organism, we can safely assume that *GGE*_*x*_*>GGE*_*C*_ because carbon is always partially lost in CO_2_. The actual values of *GGE* for carbon and other elements depend on the growth rate, the amount of each element available in food (*C*:*X* ratio), the possible absorption efficiency, and the body mass (due to metabolic rate allometry). Without information about these relationships, it can only be assumed that the gross growth efficiency of an essential nutritional element (*GGE*_*x*_) may be maximized. Regardless, a larger difference between *GGE*_*x*_ and *GGE*_*C*_ indicates a more severe trophic mismatch. Based on the limited amount of published data (see [8]), we assumed that a minimum value for *GGE*_*C*_ in an invertebrate would not exceed approximately 0.25, whereas the maximum possible *GGE*_*x*_ could approach 1 (although its actual value may be lower). Given these assumptions, the minimum balanced ratio (*GGE*_*x*_*/GGE*_*C*_) may reach a value of 1/0.25 = 4. Assuming a lower *GGE*_*C*_ (e.g., 0.1, with 90% of assimilated carbon being excreted in respiration, which is unlikely), we would obtain a threshold value of 10. With a lower maximum *GGE*_*x*_ value (e.g., 0.5) and *GGE*_*C*_ = 0.25, the threshold may be as low as 2.0, and all of the values remain within the same order of magnitude. Thus, to obtain insight into the approximate stoichiometric mismatch, we can conservatively assume that for *TSR*_*x*_*≥*4.0, the element *x* may impose a constraint on growth, with more severe mismatches indicated by even higher values. *TSR* is not meant to represent the actual measured *TER* of a given element, but it serves as a relative index indicating potential stoichiometric mismatch. Various elements may be differentially acquired, assimilated, reused, and excreted. As the *TSR* index assumes that non-carbon elements in food are assimilated by the body at a maximal rate (100%), the actual mismatches in natural situations may only be greater than the estimated *TSR* values.

We calculated *TSR*_*x*_ as follows:

$$TSR_{x}={\left(C:X\right)}_{food}/{\left(C:X\right)}_{consumer}$$

where C represents the carbon concentration and *X* represents the concentration of element *x*.

The *TSR* index does not depend on the units that are used for the stoichiometric ratios of *C*:*X* (molar or mass units). A *TSR*_*x*_*≥*4 indicates a stoichiometric mismatch that limits the development of the considered organism, with more severe mismatches indicated by higher values.

The *TSR* index is based on the separately estimated chemical compositions of insect bodies and pollen. Moreover, the analytical procedures require separate sample preparations for the CHNS analyzer and the other methods used. Due to the small size of the insects and the difficulty in obtaining a perfect homogenate, we used entire specimens (or samples of several specimens). Because the C, N and S contents were analyzed in different specimens from those used for the other elements, a direct comparison of the *TSR* indices among various experimental groups using analysis of variance (ANOVA) was not possible. Therefore, we applied a randomization procedure and calculated the *TSR* values from the *C*:*X* ratios of randomly drawn values from the distributions of measured element contents for bees and pollen. The number of possible recombined *TSR* values in various groups could reach approximately 3,000 to 20,000, of which 3,000 *TSR* values for each *C*:*X* ratio were drawn.

The Kruskal-Wallis test was used to assess the significance (p<0.05) of differences among the *TSR* values calculated for every possible combination of caste/*PPM* and sex/*PPM* for a single element. Therefore, 22 separate analyses (11 for *PPMs* 1, 1A-C; and 11 for *PPMs* 2, 2A-D) were performed, for N, P, S, K, Na, Ca, Mg, Fe, Zn, Mn, and Cu, each considered separately. This approach allowed for conclusions concerning the possible differences in *TSR* values between (1) mixed *PPMs* and the corresponding sorted *PPMs* and (2) between different castes and sexes.

To assess the possibility of limitations imposed on the growth and development of bees by the compositions of utilized pollen from various species, the *PPMs* were divided into 4 groups based on the variations in the calculated *TSR* values: (1) limiting–more than 75% of calculated *TSR*≥4; (2) likely limiting– 50–75% of calculated *TSR*≥4; (3) possibly limiting– 25–49% of calculated *TSR*≥4; and (4) non-limiting–more than 75% of calculated *TSR*<4. This grouping was performed for every element separately to indicate the set of elements that, due to their scarcity in pollen, potentially co-limited the growth and development of bees.

#### Other statistical analyses

A principal component analysis (PCA, Canoco 5) was used to compare the multi-elemental stoichiometric relations among bee castes/sexes. The data were log-transformed, centered and standardized by PCA species but not by PCA samples; thus, PCA was performed on a correlation matrix. To assess differences among the indicated clusters, we computed ANOVAs independently for the 1^st^ and 2^nd^ axis scores. We used ANOVA (Statistica 10) to assess the significance (p<0.05) of differences among castes/sexes and among *PPMs* in elemental composition.

The complete data are presented in the supplement.

### Research task (2): Literature review

We used the available data on the multi-elemental contents of various bee-collected pollen pellets and hand-collected pollen (23 studies containing data collected worldwide [23–25,34–50]) to study (1) variability in the elemental composition of pollen according to plant taxon and (2) differences in the nutritional quality between pollen from single species, genera, or families and polyfloral pollen (obtained from various taxa).

For every individual study, we considered the mean measured concentration of an element for a single taxon (species, genus, family or polyfloral pollen) per genotype and per collection site (see details in supplementary S6 Table). We calculated the variability in the concentration of various elements as the *maximum*:*minimum* ratio of the reported mean concentrations, standard deviations and coefficients of variation.

Additionally, we calculated the possible limiting effects on the growth and development of bees by utilizing pollen from various plant taxa. To that end, we calculated the *TSR* values using the reported mean concentrations of non-C elements in pollen (C concentrations were not reported), the mean C concentration in pollen measured in this study (48.6% d.m.), and the mean concentrations of elements in the bee bodies measured in this study. Based on the calculated *TSR* values, we identified pollen taxa that may be (1) stoichiometrically well balanced for bees and may promote their growth and development (for which the concentrations of at least 9 elements were reported in a single study, and none of them apart from Na had TSR≥4; we ignored Na limitations because in this study, we found that bees may not be able to gather the necessary amount of Na from pollen and should obtain Na from other sources) and taxa that may be (2) stoichiometrically highly unbalanced and thus may greatly limit the growth and development of bees (for which the concentrations of at least 9 elements were reported in a single study, and at least 3 of these elements were found to have TSR≥4 in 2 or 3 bee castes/sexes).

## Results: Research task (1)

### Body compositions of honeybee castes and sexes

#### Relative contents of elements and stoichiometry

The mean C concentration ranged from 46 to 48% d.m. and differed significantly among castes (queens and drones>workers, Table 1). The mean N concentrations ranged from 11 to 12% d.m., with queens having a significantly increased nitrogen content (Table 1). Compared with females, drones had a significantly lower P concentration (Table 1). Significant differences in body element contents were also found for minor elements. Queens were the richest in Zn, and workers were richer in Zn than were drones. Queens had higher concentrations of S, Na, Fe and Mn and lower concentrations of K and Cu than did individuals of the other castes and sex, and drones exhibited the lowest Mg and Ca levels.

**Table 1:** Average concentrations of elements in honeybee castes and sexes.

		C	N	P	S	K	Na	Ca	Mg	Fe	Zn	Mn	Cu
		% d.m.					ppm d.m.						
Queens	Mean	48.44	11.89	1.17	0.68	1.51	1045.88	521.10	1298.51	109.63	105.54	3.86	21.27
	SD	1.17	0.99	0.17	0.05	0.12	107.23	66.81	159.81	15.06	9.05	0.72	5.11
Workers	Mean	46.36	10.80	1.12	0.60	1.68	711.12	508.78	1216.98	87.25	84.08	3.07	25.17
	SD	0.72	0.32	0.17	0.06	0.11	63.89	55.04	82.74	7.34	6.50	0.40	2.46
Drones	Mean	47.93	11.23	0.94	0.57	1.63	719.63	365.56	960.53	86.72	74.09	3.08	25.22
	SD	1.40	0.56	0.23	0.05	0.10	84.85	42.58	92.60	12.24	6.85	0.36	2.13
Difference patterns		Q = D>W	Q>W = D	Q = W>D	Q>W = D	Q<W = D	Q>W = D	Q = W>D	Q = W>D	Q>W = D	Q>W>D	Q>W = D	Q<W = D
p		= 0.00003	= 0.00027	= 0.01199	<0.00001	= 0.00149	<0.00001	<0.00001	<0.00001	= 0.00002	<0.00001	= 0.00008	= 0.00353
df factor, df error		2, 44	2, 44	2, 37	2, 44	2, 40	2, 43	2, 43	2, 42	2, 38	2, 39	2, 44	2, 43
F		13.59	9.98	5.00	16.43	7.69	72.64	36.67	35.27	14.37	59.96	11.78	6.46

Difference patterns (between castes/sexes): =, no significant difference; < and >, directions of significant differences between caste/sex categories (one-way ANOVA, p < 0.05); Q, queens; W, workers; D, drones. See S1 Table for details.

**Fig 2:**
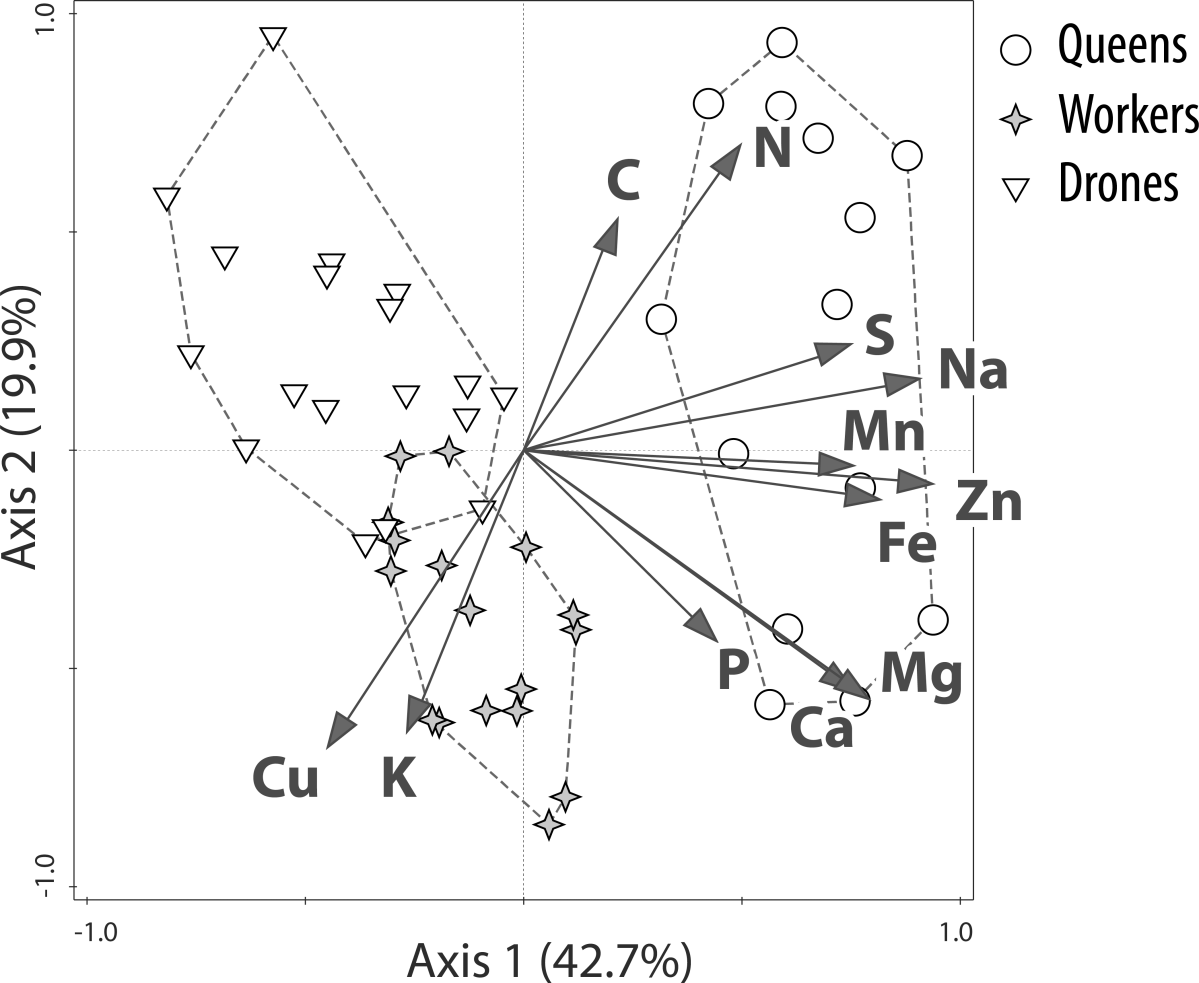
PCA plot—Multivariate analysis of stoichiometric relations in honeybee castes and sexes. The first two axes are presented. Queens are separated from the other castes/sexes primarily due to their relatively high concentrations of Zn, Na, Fe, Mg, Ca, Mn and S. Drones form a cluster separate from all females mainly due to the males’ relatively high concentrations of C and N and low concentrations of P, Ca, Mg and Zn. These tendencies were confirmed by ANOVA computed independently for the 1^st^ and 2^nd^ axis scores (p<0.05).

We compared the multi-elemental stoichiometries of the different castes and sexes using PCA. On the plane determined by the first two axes (62.6% of the total variance), the bees formed groups according to caste and sex (Fig 2). The 1^st^ component was primarily loaded by the variance in Zn (loading: 0.91), Na (0.88), Fe (0.79), Mg (0.76), Ca (0.73), Mn (0.73), and S (0.73), whereas the 2^nd^ component was primarily loaded by N (0.68), Cu (-0.66), K (-0.62), Mg (-0.55), Ca (-0.53) and C (0.51) (cf. Fig 2). The queens constituted a separate cluster, which was shifted relative to the drones and workers mainly along the 1^st^ axis (Fig 2). The drone cluster only slightly overlapped with and was shifted relative to the worker cluster along both the 1^st^ and 2^nd^ axes. The drone cluster was shifted in relation to the clusters of females due to the relatively low concentrations of P, Mg, Ca and Zn (Fig 2). These tendencies were confirmed by ANOVA computed independently for the 1^st^ and 2^nd^ axis scores (p<0.05).

Thus, all three castes and sexes were built according to specific elemental requirements: queens differed from the drones and workers mainly due to the queens’ higher concentrations of Zn, Na and Fe, whereas drones had especially low concentrations of Mg, Ca and P. The multi-elemental stoichiometry of the workers placed them between the drones and queens along the 1^st^ axis. The drones’ stoichiometry was similar to queens and differed from workers based on the 2^nd^ axis.

#### Absolute contents of elements

Among the castes and sexes, drones were the richest in the absolute contents of C and N masses in their bodies (mean values of 28 and 6 mg, respectively; Table 2). Queens were significantly poorer in C and N contents, and workers contained the smallest amounts of these elements (mean values of 9 and 2 mg, respectively; Table 2). The bodies of workers contained the smallest amounts of all of the elements. Queens and drones differed significantly in the total amounts of some minor elements. Queens were the richest caste/sex in Na, Mg, Ca, and Zn, whereas drones were the richest caste/sex in K and Cu. The total amounts of S, Mn and Fe did not differ between these two sexes (Table 2).

**Table 2:** Average total amounts of elements in honeybee castes and sexes.

		C	N	P	S	K	Na	Ca	Mg	Fe	Zn	Mn	Cu
		mg								μg			
Queens	Mean	22.074	5.387	0.548	0.308	0.698	0.048	0.024	0.059	5.091	4.792	0.177	0.972
	SD	2.701	0.490	0.071	0.030	0.042	0.005	0.002	0.006	0.452	0.450	0.036	0.205
Workers	Mean	9.084	2.114	0.208	0.118	0.334	0.014	0.010	0.024	1.734	1.671	0.061	0.505
	SD	0.838	0.170	0.071	0.015	0.022	0.001	0.001	0.001	0.144	0.149	0.007	0.056
Drones	Mean	27.675	6.494	0.531	0.330	0.915	0.040	0.021	0.054	4.796	4.153	0.172	1.416
	SD	4.300	1.124	0.135	0.064	0.053	0.003	0.002	0.004	0.526	0.291	0.015	0.162
Difference patterns		D>Q>W	D>Q>W	D = Q>W	D = Q>W	D>Q>W	Q>D>W	Q>D>W	Q>D>W	D = Q>W	Q>D>W	D = Q>W	D>Q>W
P		<0.00001	<0.00001	<0.00001	<0.00001	<0.00001	<0.00001	<0.00001	<0.00001	<0.00001	<0.00001	<0.00001	<0.00001
df factor, df error		2, 44	2, 44	2, 37	2, 44	2, 40	2, 43	2, 43	2, 42	2, 38	2, 39	2.44	2, 43
F		161.12	151.73	46.56	115.06	853.17	410.00	242.57	342.75	270.82	421.33	146.04	144.85

Difference patterns: significant differences between caste/sex categories (one-way ANOVA, p<0.05); Q, queens; W, workers; D, drones. Detailed results are presented in S2 Table.

### Species compositions of pollen pellet morphospecies (*PPMs*)

The *PPMs* differed in pollen species composition and consisted of 6 (*PPM2A*) to 18 (*PPMs* 1B and 1C) pollen species. In the majority of *PPMs*, one species dominated, and in 7 of 9 *PPMs*, the dominant species constituted more than 40% of all of the pollen grains, whereas the next most dominant species constituted less than 30%. Three of the *PPMs* (nos. 1A, 2A, and 2C) had one species composing more than 70% of the pollen grains. The main taxa composing the *PPMs* are shown in Table 3, and the detailed data are presented in S3 Table.

**Table 3:** Main (>16%) taxa composing the studied *PPMs*. Bold text indicates pollen with more than 70% of the contents obtained from a single species. Detailed data are presented in S3 Table. Mixed *PPMs* 1 and 2 constituted the pool of pollen that was collected by the honeybees. Sorted *PPMs* (1A-C sorted from 1 and 2A-D sorted from 2) had specific species compositions that differed from those of mixed *PPMs* but contributed to those of mixed *PPMs*.

*PPM* No.	Main plant taxa found in the pollen samples	Pollen grain content [%]	Number of taxa included in the pollen samples
1	*Prunus*	51	17
	*Brassica napus*	18	
**1A**	***Prunus***	**71**	**13**
1B	*Anthriscus*	21	18
	*Aesculus*	20	
1C	*Salix*	42	18
	*Brassica napus*	29	
2	*Trifolium repens*	37	15
	*Brassica napus*	24	
	*Filipendula*	20	
**2A**	***Trifolium repens***	**87**	**6**
2B	*Rhus typhina*	61	12
	*Trifolium repens*	18	
**2C**	***Pyrus***	**73**	**10**
2D	*Filipendula*	58	7
	*Trifolium repens*	19	
	*Brassica napus*	18	

### Elemental contents of *PPMs* and nectar

**Fig 3:**
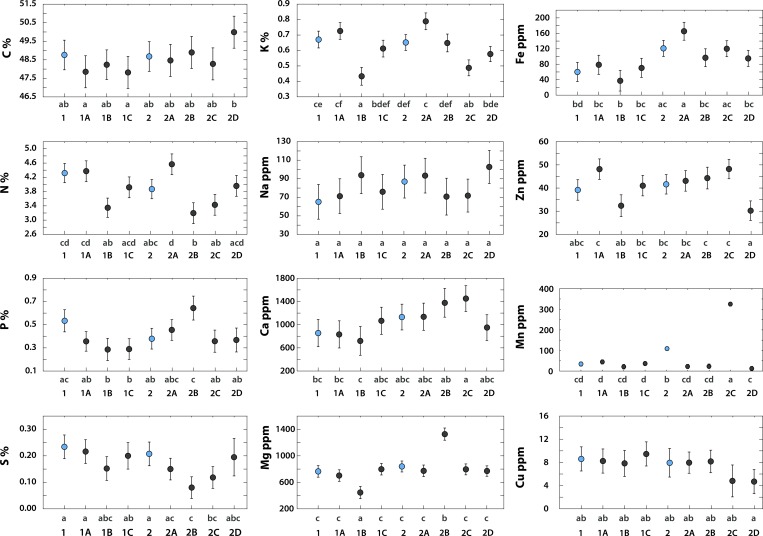
Concentrations of elements in honeybee pollen pellets (Means and CI). ANOVA, p<0.05. Various lowercase letters denote statistically significant differences. Mixed pollen pellets (polyfloral, as they were gathered by bees) are shown in blue and sorted pellets (sorted from mixed pollen pellets, which had varying compositions of pollen from different species but contributed to the compositions of mixed pollen pellets) are shown in gray. Commercial pollen pellets: mixed, 1; sorted, 1A to 1C. Study site pollen pellets: mixed, 2; sorted, 2A to 2D. See text for explanation. Whiskers denote 95% confidence intervals. Detailed results are presented in S4 Table. Sorted pollen pellets exhibited greater variance in the element concentrations; mixed pollen pellets tended to have average concentrations of the studied elements.

The study site pollen pellets did not differ significantly from the commercial pellets in the concentrations of any nutritional elements except Fe and Mn (Fig 3). The sorted *PPMs* showed greater variance in the element concentrations than did the mixed *PPMs* (Fig 3). The largest differences among *PPMs* in nutritional element concentrations were observed for Mn, Fe, Mg, S, Cu and P. The C content in all of the *PPMs* was less than 50%. The N content ranged between 3 and 4.5%, and the P content ranged between 0.3 and 0.6%. A high content of clover pollen (99% *T*. *repens*+*T*. *pratense* in *PPM2A)* resulted in the highest concentrations of N, K and Fe (Fig 3). Differences in taxonomic composition among *PPMs* tended to be reflected in their different elemental compositions (Fig 3). Nectar showed extremely low concentrations of all non-carbon elements (Table 4).

**Table 4:** Mineral composition of nectar stored in beehives. Mean values were calculated for 24 samples collected from 5 beehives.

	P	K	Na	Ca	Mg	Fe	Zn	Mn	Cu
	mg/l						μg/l		
Mean	0.064	1.191	0.025	0.104	0.149	0.002	1.948	0.892	0.290
SD	0.034	0.478	0.008	0.013	0.026	0.002	2.353	0.594	0.086

### Stoichiometric mismatch expressed as the trophic stoichiometric ratio (*TSR*)

**Fig 4:**
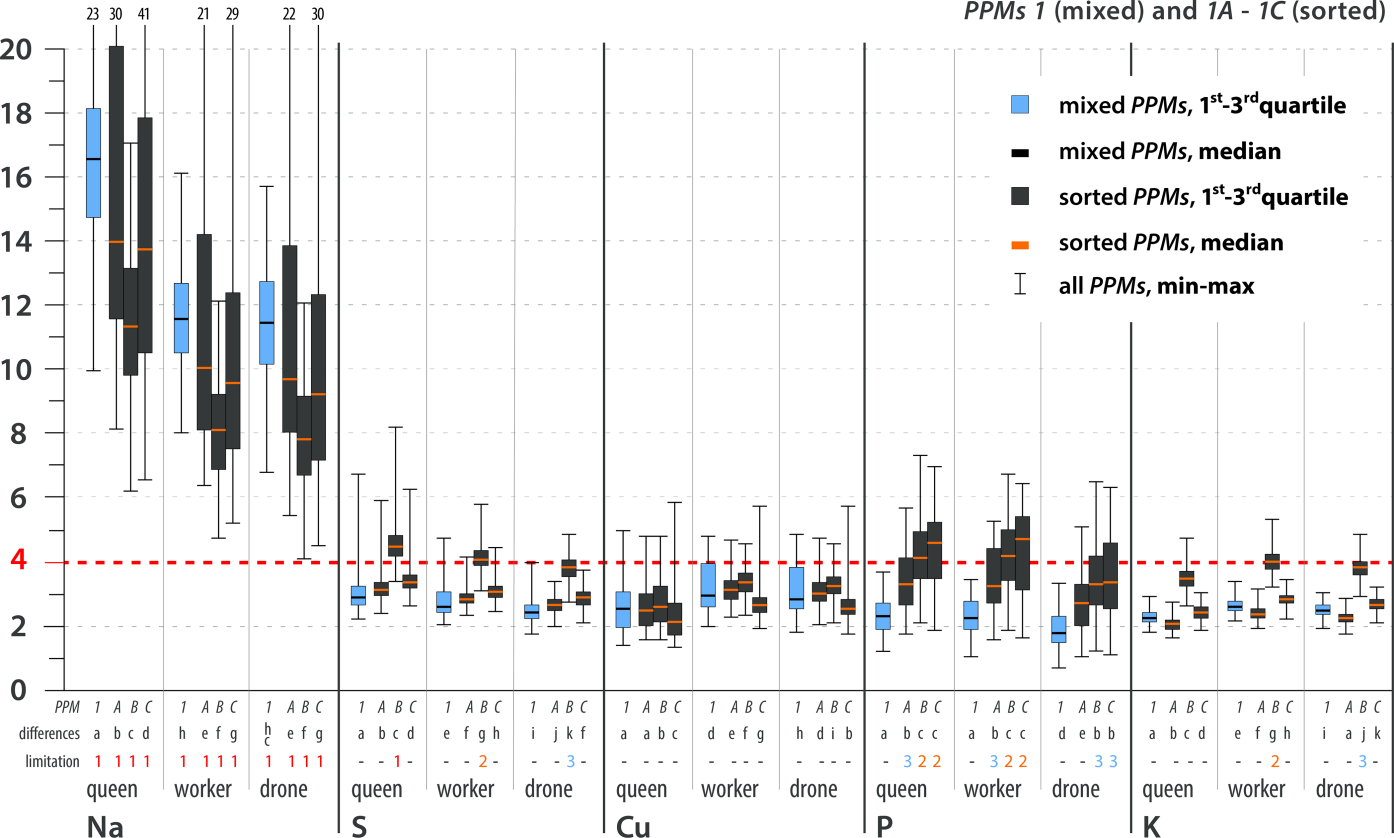
Trophic stoichiometric ratios (*TSR = (C*:*X)Pollen Pellets/(C*:*X)Bee*; *C* = Carbon Concentration, *X* = Concentration of element *x*) for the studied elements. **Most limiting elements, *PPM*1.** TSR values ≥4 denote limitations on growth and development. The dotted red line indicates the threshold value of TSR = 4. Limitation symbols (**1, 2, 3, -**) denote the possibility of a limitation of bee growth and development due to the scarcity of a certain element in pollen. Calculated *TSR* values were grouped into 4 categories: (**1**) limiting–more than 75% of calculated *TSR* values ≥4; (**2**) likely limiting– 50–75% of calculated *TSR* values ≥4; (**3**) possibly limiting– 25–49% of calculated *TSR* values ≥4; (-) non-limiting–more than 75% of calculated *TSR* values <4. *PPM* = pollen pellet morphospecies. For every element and caste, *PPMs* are placed with 1 at the farthest left, followed by 1A-C, 2 and 2A-D (farthest right). Mixed *PPMs* 1 and 2 = pollen pellets gathered by honeybees; sorted *PPMs* = pollen pellets of different pollen species composition that were sorted from the mixed *PPMs*. *PPMs* categories are explained in the text. For each element, considered separately, various lowercase letters denote statistically significant differences (indicated as “differences” in the figure). Kruskal-Wallis test, p<0.001; for every single element: for *PPM*1 12 grouping variables, N = 36000, for PPM2 15 grouping variables, N = 45000. See text for an explanation of the *TSR* calculation and S5 Table for detailed results. All of the *PPMs* limit bee development due to Na scarcity. Concerning other elements, mixed *PPMs* tend not to be limiting for bees, while sorted *PPMs* tend to be limiting to varying degrees. The possibility of co-limitation by scarcities of S, Cu, P, K, Zn or N depends on *PPM*, species composition, and caste/sex.

**Fig 5:**
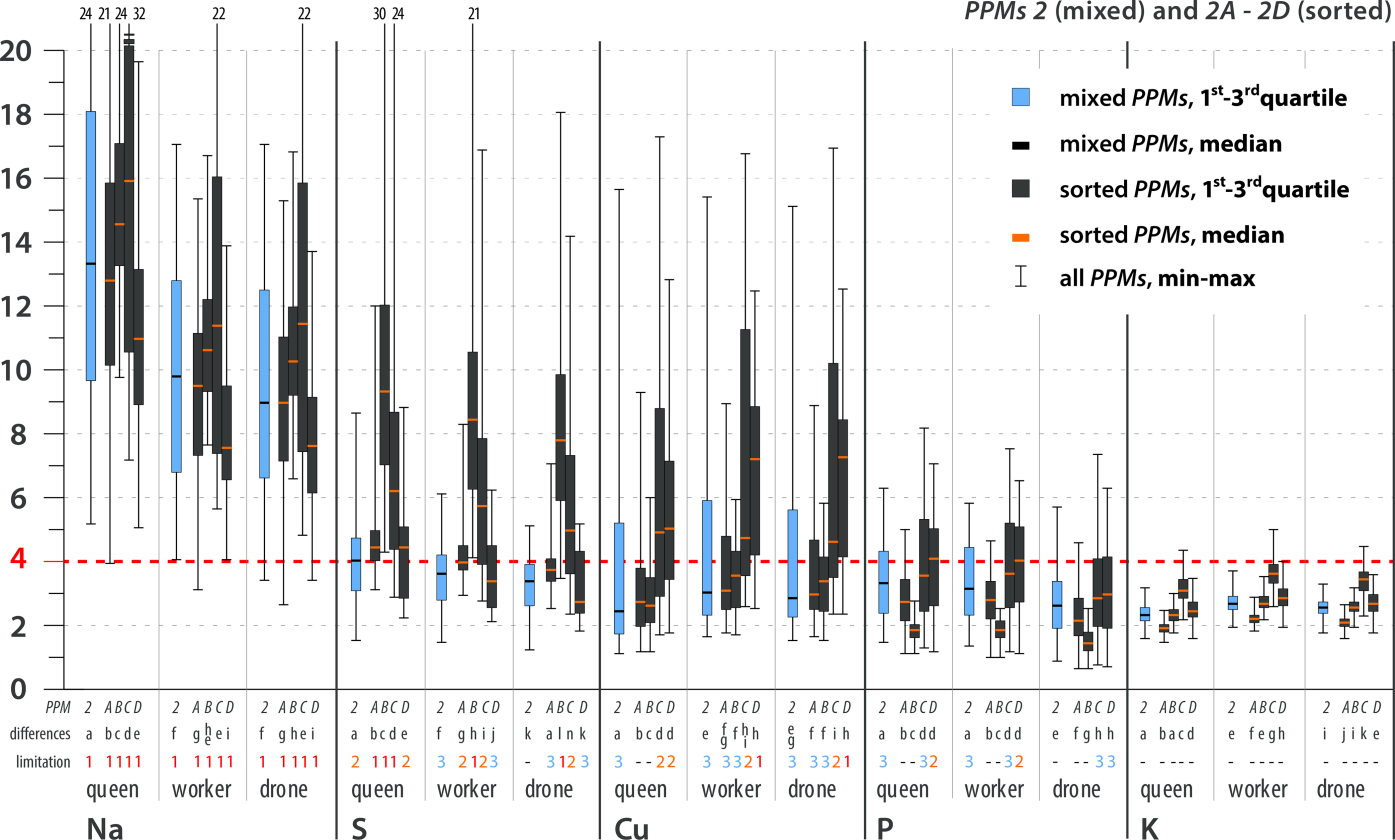
Trophic stoichiometric ratios (*TSR = (C*:*X)Pollen Pellets/(C*:*X)Bee*; *C* = Carbon Concentration, *X* = Concentration of element *x*) for the studied elements. **Most limiting elements, *PPM*2.** TSR values ≥4 denote limitations on growth and development. The dotted red line indicates the threshold value of TSR = 4. Limitation symbols (**1, 2, 3, -**) denote the possibility of a limitation of bee growth and development due to the scarcity of a certain element in pollen. Calculated *TSR* values were grouped into 4 categories: (**1**) limiting–more than 75% of calculated *TSR* values ≥4; (**2**) likely limiting– 50–75% of calculated *TSR* values ≥4; (**3**) possibly limiting– 25–49% of calculated *TSR* values ≥4; (-) non-limiting–more than 75% of calculated *TSR* values <4. *PPM* = pollen pellet morphospecies. For every element and caste, *PPMs* are placed with 1 at the farthest left, followed by 1A-C, 2 and 2A-D (farthest right). Mixed *PPMs* 1 and 2 = pollen pellets gathered by honeybees; sorted *PPMs* = pollen pellets of different pollen species composition that were sorted from the mixed *PPMs*. *PPMs* categories are explained in the text. For each element, considered separately, various lowercase letters denote statistically significant differences (indicated as “differences” in the figure). Kruskal-Wallis test, p<0.001; for every single element: for *PPM*1 12 grouping variables, N = 36000, for PPM2 15 grouping variables, N = 45000. See text for an explanation of the *TSR* calculation and S5 Table for detailed results. All of the *PPMs* limit bee development due to Na scarcity. Concerning other elements, mixed *PPMs* tend not to be limiting for bees, while sorted *PPMs* tend to be limiting to varying degrees. The possibility of co-limitation by scarcities of S, Cu, P, K, Zn or N depends on *PPM*, species composition, and caste/sex.

**Fig 6:**
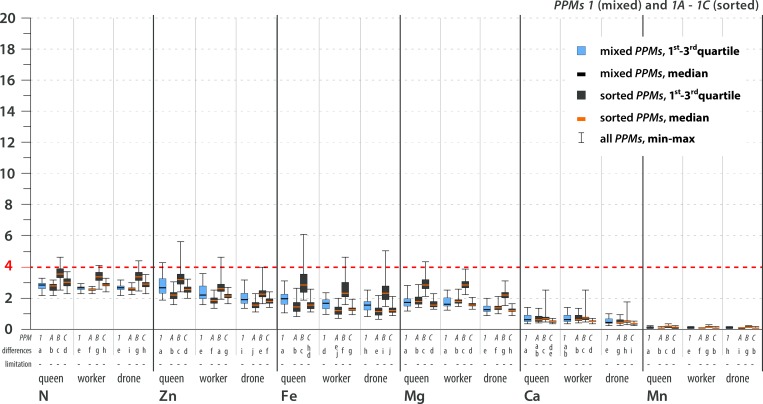
Trophic stoichiometric ratios (*TSR = (C*:*X)Pollen Pellets/(C*:*X)Bee*; *C* = Carbon Concentration, *X* = Concentration of Element *x*) for the studied elements. **Possibly limiting and non-limiting elements, *PPM*1.** TSR values ≥4 denote limitations on growth and development. The dotted red line indicates the threshold value of TSR = 4. Limitation symbols (**1, 2, 3, -**) denote the possibility of a limitation of bee growth and development due to the scarcity of a certain element in pollen. Calculated *TSR* values were grouped into 4 categories: (**1**) limiting–more than 75% of calculated *TSR* values ≥4; (**2**) likely limiting– 50–75% of calculated *TSR* values ≥4; (**3**) possibly limiting– 25–49% of calculated *TSR* values ≥4; (-) non-limiting–more than 75% of calculated *TSR* values <4. *PPM* = pollen pellet morphospecies. For every element and caste, *PPMs* are placed with 1 at the farthest left, followed by 1A-C, 2 and 2A-D (farthest right). Mixed *PPMs* 1 and 2 = pollen pellets gathered by honeybees; sorted *PPMs* = pollen pellets of different pollen species composition that were sorted from the mixed *PPMs*. *PPMs* categories are explained in the text. For each element, considered separately, various lowercase letters denote statistically significant differences (indicated as “differences” in the figure). Kruskal-Wallis test, p<0.001; for every single element: for *PPM*1 12 grouping variables, N = 36000, for PPM2 15 grouping variables, N = 45000. See text for an explanation of the *TSR* calculation and S5 Table for detailed results. All of the *PPMs* limit bee development due to Na scarcity. Concerning other elements, mixed *PPMs* tend not to be limiting for bees, while sorted *PPMs* tend to be limiting to varying degrees. The possibility of co-limitation by scarcities of S, Cu, P, K, Zn or N depends on *PPM*, species composition, and caste/sex.

**Fig 7:**
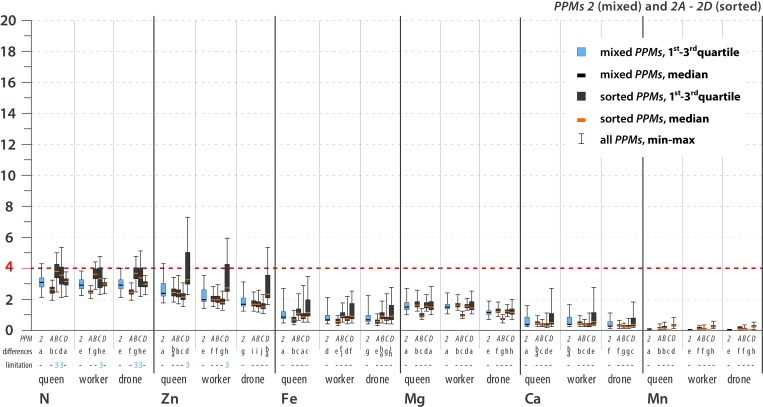
Trophic stoichiometric ratios (*TSR = (C*:*X)Pollen Pellets/(C*:*X)Bee*; *C* = Carbon Concentration, *X* = Concentration of Element *x*) for the studied elements. **Possibly limiting and non-limiting elements, *PPM*2.** TSR values ≥4 denote limitations on growth and development. The dotted red line indicates the threshold value of TSR = 4. Limitation symbols (**1, 2, 3, -**) denote the possibility of a limitation of bee growth and development due to the scarcity of a certain element in pollen. Calculated *TSR* values were grouped into 4 categories: (**1**) limiting–more than 75% of calculated *TSR* values ≥4; (**2**) likely limiting– 50–75% of calculated *TSR* values ≥4; (**3**) possibly limiting– 25–49% of calculated *TSR* values ≥4; (-) non-limiting–more than 75% of calculated *TSR* values <4. *PPM* = pollen pellet morphospecies. For every element and caste, *PPMs* are placed with 1 at the farthest left, followed by 1A-C, 2 and 2A-D (farthest right). Mixed *PPMs* 1 and 2 = pollen pellets gathered by honeybees; sorted *PPMs* = pollen pellets of different pollen species composition that were sorted from the mixed *PPMs*. *PPMs* categories are explained in the text. For each element, considered separately, various lowercase letters denote statistically significant differences (indicated as “differences” in the figure). Kruskal-Wallis test, p<0.001; for every single element: for *PPM*1 12 grouping variables, N = 36000, for PPM2 15 grouping variables, N = 45000. See text for an explanation of the *TSR* calculation and S5 Table for detailed results. All of the *PPMs* limit bee development due to Na scarcity. Concerning other elements, mixed *PPMs* tend not to be limiting for bees, while sorted *PPMs* tend to be limiting to varying degrees. The possibility of co-limitation by scarcities of S, Cu, P, K, Zn or N depends on *PPM*, species composition, and caste/sex.

The stoichiometric mismatch (i.e., the constraint on growth and development) was represented by the calculated *TSR* values and differed by element, *PPM*, caste and sex (Figs 4–7; see S5 Table for details). The variance in *TSR* values was higher for the sorted *PPMs* than for the mixed *PPMs* (compared with mixed *PPMs*, sorted *PPMs* showed both relatively high and relatively low *TSR* values). The sorted *PPMs* were more likely than the mixed *PPMs* to limit bee development. The mixed pollen and all sorted *PPMs* were limiting for all castes due to Na scarcity. S and Cu were limiting or likely limiting for all castes in some sorted *PPMs*; K was likely limiting for workers and possibly limiting for drones in one sorted *PPM*; P was likely limiting for females in some sorted *PPMs*, possibly limiting for all castes in sorted *PPMs*, and possibly limiting for females in mixed *PPM2*; and N (for all castes in some sorted *PPMs*) and Zn (for females in sorted *PPM2D*) were possibly limiting elements (Table 5; see Figs 4–7 for statistical analysis). In general, limiting or likely limiting effects were observed with respect to Na, S, Cu, P and K (Table 5); therefore, the growth and development of honeybees may be co-limited mainly by these five elements, but possibly also by N and Zn (Table 5; see Figs 4–7 for statistical analysis). Differences in *TSR* values between castes and sexes were observed considering limiting, likely limiting and possibly limiting elements, as follows: S (queen>worker>drone), K (worker>drone>queen), Zn (queen>worker>drone), Na (queen>worker = drone), Cu (worker≥drone>queen), N (queen>worker = drone). Differences in *TSR* values between sexes were observed for P (queen = worker>drone) (Table 5; see Figs 4–7 for statistical analysis).

**Table 5:** Limiting effect of mixed (1, 2) and sorted (1A-C, 2A-D) pollen pellets on various honeybee castes and sexes. Symbols (**1, 2, 3, -**) denote the possibility of a limitation on bee growth and development due to the scarcity of a certain element in pollen. *TSR* values ≥4 denote limitations on growth and development. Calculated *TSR* values were grouped into 4 categories: (**1**) limiting–more than 75% of calculated *TSR* values ≥4; (**2**) likely limiting– 50–75% of calculated *TSR* values ≥4; (**3**) possibly limiting– 25–49% of calculated *TSR* values ≥4; (-) non-limiting–more than 75% of calculated *TSR* values <4. The growth and development of bees may be co-limited by Na, S, Cu, P and K; limitation based on N and Zn may also be possible.

PPM	Caste	N	P	S	K	Na	Ca	Mg	Fe	Zn	Mn	Cu
1	queen	**-**	**-**	**-**	**-**	**1**	**-**	**-**	**-**	**-**	**-**	**-**
1A	queen	**-**	**3**	**-**	**-**	**1**	**-**	**-**	**-**	**-**	**-**	**-**
1B	queen	**-**	**2**	**1**	**-**	**1**	**-**	**-**	**-**	**-**	**-**	**-**
1C	queen	**-**	**2**	**-**	**-**	**1**	**-**	**-**	**-**	**-**	**-**	**-**
1	worker	**-**	**-**	**-**	**-**	**1**	**-**	**-**	**-**	**-**	**-**	**-**
1A	worker	**-**	**3**	**-**	**-**	**1**	**-**	**-**	**-**	**-**	**-**	**-**
1B	worker	**-**	**2**	**2**	**2**	**1**	**-**	**-**	**-**	**-**	**-**	**-**
1C	worker	**-**	**2**	**-**	**-**	**1**	**-**	**-**	**-**	**-**	**-**	**-**
1	drone	**-**	**-**	**-**	**-**	**1**	**-**	**-**	**-**	**-**	**-**	**-**
1A	drone	**-**	**-**	**-**	**-**	**1**	**-**	**-**	**-**	**-**	**-**	**-**
1B	drone	**-**	**3**	**3**	**3**	**1**	**-**	**-**	**-**	**-**	**-**	**-**
1C	drone	**-**	**3**	**-**	**-**	**1**	**-**	**-**	**-**	**-**	**-**	**-**
2	Queen	**-**	**3**	**2**	**-**	**1**	**-**	**-**	**-**	**-**	**-**	**3**
2A	Queen	**-**	**-**	**1**	**-**	**1**	**-**	**-**	**-**	**-**	**-**	**-**
2B	Queen	**3**	**-**	**1**	**-**	**1**	**-**	**-**	**-**	**-**	**-**	**-**
2C	Queen	**3**	**3**	**1**	**-**	**1**	**-**	**-**	**-**	**-**	**-**	**2**
2D	Queen	**-**	**2**	**2**	**-**	**1**	**-**	**-**	**-**	**3**	**-**	**2**
2	worker	**-**	**3**	**3**	**-**	**1**	**-**	**-**	**-**	**-**	**-**	**3**
2A	worker	**-**	**-**	**2**	**-**	**1**	**-**	**-**	**-**	**-**	**-**	**3**
2B	worker	**-**	**-**	**1**	**-**	**1**	**-**	**-**	**-**	**-**	**-**	**3**
2C	worker	**3**	**3**	**2**	**-**	**1**	**-**	**-**	**-**	**-**	**-**	**2**
2D	worker	**-**	**2**	**3**	**-**	**1**	**-**	**-**	**-**	**3**	**-**	**1**
2	drone	**-**	**-**	**-**	**-**	**1**	**-**	**-**	**-**	**-**	**-**	**3**
2A	drone	**-**	**-**	**3**	**-**	**1**	**-**	**-**	**-**	**-**	**-**	**3**
2B	drone	**3**	**-**	**1**	**-**	**1**	**-**	**-**	**-**	**-**	**-**	**3**
2C	drone	**3**	**3**	**2**	**-**	**1**	**-**	**-**	**-**	**-**	**-**	**2**
2D	drone	**-**	**3**	**3**	**-**	**1**	**-**	**-**	**-**	**-**	**-**	**1**

## Results: Research task (2)

### Elemental content of pollen according to data in the literature

The data in the literature demonstrated great variation in the element concentrations of pollen, which appeared to result from taxonomic differences in pollen stoichiometry. Concerning the mean element concentrations in various pollen taxa, Mn, Na and Fe and Zn were the most variable, with Cu, Ca, K and P also demonstrating high variability (Table 6, see S6 Table for details).

**Table 6:** Variability, ranges and mean values of elemental concentrations in pollen of various taxa (one species, one genus, one family, polyfloral). Information is based on data in the literature collected worldwide.

	N	P	S	K	Na	Ca	Mg	Fe	Zn	Mn	Cu
	% d.m.				ppm d.m.						
Mean	5.68	0.50	0.27	0.55	173	1710	1186	96	72	49	13
N	14	125	71	167	131	175	173	189	195	149	175
Min	2.20	0.05	0.11	0.13	5	300	216	6	16	5	2
Max	7.36	1.16	0.45	3.80	1549	11,800	3800	808	795	685	59
Max/Min	3	23	4	29	313	39	18	135	50	137	34
SD	1.61	0.23	0.09	0.33	251	1371	719	124	78	73	10
c.v.	0.28	0.45	0.33	0.61	1.45	0.80	0.61	1.29	1.09	1.49	0.78

For each study, the mean measured concentration of an element was considered. “Min” and “Max” are the minimal and maximal mean concentrations, respectively, of the elements analyzed in all of the studied pollen taxa and polyfloral pollen. N = number of analyzed pollen taxa and polyfloral pollen loads from various taxa. Information is based on the literature review presented in detail in S6 Table.

### Stoichiometric mismatches calculated for bees utilizing various plant taxa (literature data)

Relying on the data gathered from the literature (S6 Table), we calculated the *TSR* ratios for various pollen taxa to identify which pollen taxa might be stoichiometrically well balanced for bees and may promote their growth and development (for which the concentrations of at least 9 elements were reported in a single study, and none of these elements apart from Na had TSR values ≥4). We also sought to identify taxa that may be stoichiometrically highly unbalanced, limiting the growth and development of bees to a high degree (for which at the concentrations of at least 9 elements were reported in a single study, and at least 3 of these elements had TSR values ≥4 for 2 or 3 bee castes/sexes). Stoichiometrically well-balanced taxa may include fuzzy kiwifruit (Chinese gooseberry), rough-barked apple, heath-leaved banksia, common camellia, watermelon, several species of eucalyptus, lotus, common poppy, almond, common giant mustard, magnolia-vine, clover, common gorse and broad bean (Table 7). Corn and oilseed rape were indicated as stoichiometrically well-balanced based on data reported by Yang et al. [25], though these results were not found in the other data. Buckwheat was indicated both as stoichiometrically well-balanced (based on the results of Yang et al. [25]) and highly unbalanced (based on the results of Somerville and Nicol [51]). Compared with other studies, the study by Yang et al. [25] showed relatively high concentrations of the measured elements in pollen, which indicated that a high number of pollen taxa were stoichiometrically well-balanced (Table 7). Todd and Bretherick [50] reported very low concentrations of Fe in all the studied taxa, indicating limiting effects (Table 7). These results are probably underestimated. Polyfloral pollen was indicated as well balanced stoichiometrically based on the data from 2 studies out of 12 (these limited data mainly resulted from the lack of a sufficient number of element concentrations reported in other studies). Six studies showed limiting effects of K scarcity in polyfloral pollen (Table 7). Yang et al. [25], Sommerville and Nicol [51] and Szczęsna [24] reported high concentrations of Na for all the measured pollen taxa; therefore, no limiting effects of Na were indicated based on the data from these studies (Table 7). Species with large stoichiometric imbalances may include capeweed, musk thistle, black sheoak, rush skeletonweed, catsear and lavender. Sunflower was indicated as highly stoichiometrically unbalanced based on the data of Sommerville and Nicol [51], which was not the case according to Yang et al. [25]. One of each of 12 polyfloral pollen mixes and 5 pollen samples from Salix taxa were identified as highly unbalanced (Table 7). The pollen from the remaining 50 taxa may be limiting to varying degrees due to their scarce concentrations of 1 or 2 different elements (Table 7).

**Table 7:** Limiting effects on honeybee growth and development by stoichiometry of pollen from various taxa. Calculated as *TSR* ratios (*TSR* values ≥4 denote limitations on growth and development) based on published elemental composition data on pollen from various taxa and the data from this study on C contents of pollen and the elemental composition of honeybees (mean values). The effects are caste- and sex-dependent and are indicated by symbols: Q–queen, W–worker, D–Drone; “-” no limiting effect; n.d.–no data. The limiting effect of Fe calculated in the study by Todd and Bretherick [50] is probably overestimated, as indicated by italics and “(?)”. Shaded and bolded font denotes pollen taxa that are stoichiometrically well balanced for the honeybee and shaded and underlined font indicates highly stoichiometrically unbalanced pollen taxa.

Taxon	Scarce element, limiting for indicated caste/sex											Source
	N	P	S	K	Na	Ca	Mg	Fe	Zn	Mn	Cu	
*Acacia sp*.	n.d.	-	-	-	Q; W; D	-	-	-	-	-	Q; W; D	Somerville and Nicol [51]
***Actinidia deliciosa***	-	-	-	-	Q; W; D	-	-	-	-	-	-	Clark and Lintas [41]
*Alnus glutinosa*	-	-	-	n.d.	n.d.	n.d.	n.d.	n.d.	n.d.	n.d.	n.d.	Nielsen et al. [49]
*Alnus incana*	-	Q; W	-	n.d.	n.d.	n.d.	n.d.	n.d.	n.d.	n.d.	n.d.	Nielsen et al. [49]
*Ambrosia artemisiifolia*	n.d.	n.d.	n.d.	n.d.	n.d.	n.d.	n.d.	n.d.	-	n.d.	-	Cloutier-Hurteau et al. [42]
***Angophora floribunda***	n.d.	-	-	-	Q; W; D	-	-	-	-	-	-	Somerville and Nicol [51]
*Arctotheca calendula*	n.d.	Q; W	Q; W	Q; W; D	Q; W; D	-	-	Q	-	-	-	Somerville and Nicol [51]
*Artemisia*	n.d.	n.d.	n.d.	Q; W; D	-	-		Q	-	-	W; D	Szczęsna [24]
*Asparagus officinalis*	n.d.	Q; W	n.d.	-	n.d.	-	-	-	n.d.	n.d.	n.d.	Todd and Bretherick [50]
*Asphodelus fistulosus*	n.d.	-	-	-	Q; W; D	-	-	-	-	-	Q; W; D	Somerville and Nicol [51]
***Banksia ericifolia***	n.d.	-	-	-	-	-	-	-	-	-	-	Somerville and Nicol [51]
*Brassica campestris*	n.d.	-	n.d.	-	n.d.	-	-	*Q; W; D (?)*	n.d.	n.d.	n.d.	Todd and Bretherick [50]
*Brassica kaber*	n.d.	-	n.d.	-	n.d.	-	-	*Q; W; D (?)*	n.d.	n.d.	n.d.	Todd and Bretherick [50]
*Brassica napus*	n.d.	-	-	-	Q; W; D	-	-	Q	-	-	W; D	Somerville and Nicol [51]
***Brassica napus***	n.d.	-	-	-	-	-	-	-	-	-	-	Yang et al. [25]
*Brassica nigra*	n.d.	-	n.d.	-	n.d.	-	-	*Q; W; D (?)*	n.d.	n.d.	n.d.	Todd and Bretherick [50]
*Brassicaceae*	n.d.	n.d.	n.d.	Q; W; D	Q; W; D	-	-	-	-	-	-	Kostic et al. [39]
*Brassicaceae*	n.d.	n.d.	n.d.	W; D	-	-	-	-	-	-	-	Szczęsna [24]
*Brassicaceae + Salix*	n.d.	n.d.	n.d.	Q; W; D	Q; W; D	-	-	-	-	-	-	Kostic et al. [39]
*Calandrinia ciliata*	n.d.	Q; W	n.d.	-	n.d.	-	-	*Q; W; D (?)*	n.d.	n.d.	n.d.	Todd and Bretherick [50]
*Calluna vulgaris*	n.d.	n.d.	n.d.	n.d.	n.d.	n.d.	n.d.	-	-	-	-	Paulo et al. [52]
***Camellia japonica***	n.d.	-	n.d.	-	-	-	-	-	-	-	-	Yang et al. [25]
*Carduus nutans*	n.d.	Q; W	Q; W	Q; W; D	Q; W; D	-	-	-	Q; W	-	-	Somerville and Nicol [51]
*Carthamus lanatus*	n.d.	-	-	Q; W; D	Q; W; D	-	-	-	-	-	-	Somerville and Nicol [51]
*Casuarina littoralis*	n.d.	Q; W; D	Q; W; D	Q; W; D	-	-	Q; W; D	-	Q; W; D	-	-	Somerville and Nicol [51]
*Ceanothus crassifolius*	n.d.	-	n.d.	-	n.d.	-	-	*Q; W; D (?)*	n.d.	n.d.	n.d.	Todd and Bretherick [50]
*Ceanothus integerrimus*	n.d.	Q; W	n.d.	-	n.d.	-	-	*Q; W; D (?)*	n.d.	n.d.	n.d.	Todd and Bretherick [50]
*Centaurea solstitialis*	n.d.	-	-	Q; W; D	Q; W; D	-	-	-	-	-	-	Somerville and Nicol [51]
*Centaurea solstitialis*	n.d.	Q; W	n.d.	Q; W; D	n.d.	-	-	*Q; W; D (?)*	n.d.	n.d.	n.d.	Todd and Bretherick [50]
*Chamaebatia foliolosa*	n.d.	Q; W; D	n.d.	-	n.d.	-	-	*Q; W; D (?)*	n.d.	n.d.	n.d.	Todd and Bretherick [50]
*Chondrilla juncea*	n.d.	Q; W; D	Q; W; D	Q; W; D	Q; W; D	-	Q; W	-	Q	-	Q; W; D	Somerville and Nicol [51]
*Cirsium vulgare*	n.d.	-	Q	Q; W; D	Q; W; D	-	-	-	Q	-	-	Somerville and Nicol [51]
*Cistus ladanifer*	n.d.	n.d.	n.d.	n.d.	n.d.	n.d.	n.d.	-	-	-	-	Paulo et al. [52]
*Cistus sp*.	n.d.	Q; W; D	n.d.	W	-	-	-	-	-	-	-	Bonvehi and Jordà [38]
***Citrullus lanatus***	n.d.	-	n.d.	-	-	-	-	-	-	-	-	Yang et al. [25]
*Citrus × sinensis*	n.d.	n.d.	n.d.	Q; W; D	-	-	-	-	-	-	-	Zeng and Yan [53]
*Corymbia maculata*	n.d.	-	-	-	Q; W; D	-	-	-	-	-	-	Somerville and Nicol [50]
*Cynodon dactylon*	n.d.	Q; W; D	n.d.	-	n.d.	-	-	*Q; W; D (?)*	n.d.	n.d.	n.d.	Todd and Bretherick [50]
*Dendranthema indicum*	n.d.	Q; W; D	n.d.	Q; W; D	-	-	Q	-		-	-	Yang et al. [25]
*Echium plantagineum*	n.d.	-	-	-	Q; W; D	-	-	-	-	-	Q; W; D	Somerville and Nicol [51]
***Eucalyptus albens***	n.d.	-	-	-	Q; W; D	-	-	-	-	-	-	Somerville and Nicol [51]
***Eucalyptus bridgesiana***	n.d.	-	-	-	Q; W; D	-	-	-	-	-	-	Somerville and Nicol [51]
***Eucalyptus camaldulensis***	n.d.	-	-	-	Q; W; D	-	-	-	-	-	-	Somerville and Nicol [51]
***Eucalyptus fastigata***	n.d.	-	-	-	Q; W; D	-	-	-	-	-	-	Somerville and Nicol [51]
*Eucalyptus globulus*	n.d.	-	n.d.	-	n.d.	-	-	*Q (?)*	n.d.	n.d.	n.d.	Todd and Bretherick [50]
***Eucalyptus macrorhyncha***	n.d.	-	-	-	Q; W; D	-	-	-	-	-	-	Somerville and Nicol [51]
***Eucalyptus punctata***	n.d.	-	-	-	-	-	-	-	-	-	-	Somerville and Nicol [51]
***Eucalyptus robusta***	n.d.	-	-	-	Q; W; D	-	-	-	-	-	-	Somerville and Nicol [51]
***Eucalyptus saligna***	n.d.	-	-	-	Q; W; D	-	-	-	-	-	-	Somerville and Nicol [51]
***Eucalyptus viminalis***	n.d.	-	-	-	Q; W; D	-	-	-	-	-	-	Somerville and Nicol [51]
*Fabaceae*	n.d.	n.d.	n.d.	Q; W; D	Q; W; D	-	-	-	-	-	-	Kostic et al. [39]
*Fagopyrum esculentum*	n.d.	-	Q; W; D	-	Q; W; D	-	-	-	Q; W; D	-	Q; W; D	Somerville and Nicol [51]
***Fagopyrum esculentum***	n.d.	-	n.d.	-	-	-	-	-	-	-	-	Yang et al. [25]
*Helianthus annuus*	n.d.	Q; W	Q	Q; W; D	Q; W; D	-	-	-	-	-	-	Somerville and Nicol [51]
*Helianthus annuus*	n.d.	Q; W	n.d.	W	-	-	-	-	-	-	-	Yang et al. [25]
*Helianthus annuus*	n.d.	n.d.	n.d.	Q; W; D	n.d.	-	-	Q	-	n.d.	n.d.	Stanciu et al. [37]
*Hypericum perforatum*	n.d.	-	n.d.	-	n.d.	-	-	*Q; W; D (?)*	n.d.	n.d.	n.d.	Todd and Bretherick [50]
*Hypochoeris radicata*	n.d.	Q; W; D	Q; W; D	Q; W; D	Q; W; D	-	Q; W	Q; W; D	Q; W	-	Q; W; D	Somerville and Nicol [51]
*Juglans nigra*	n.d.	-	n.d.	-	n.d.	-	-	-	n.d.	n.d.	n.d.	Todd and Bretherick [50]
*Juglans regia*	n.d.	n.d.	n.d.	-	Q; W; D	-	-	-	-	-	-	Cosmulescu et al. [43]
*Lavandula sp*.	n.d.	Q; W	-	Q; W; D	Q; W; D	-	Q; W	Q; W; D	Q; W; D	-	-	Somerville and Nicol [50]
*Legume*	n.d.	Q; W; D	n.d.	W; D	n.d.	-	-	-	n.d.	n.d.	n.d.	Todd and Bretherick [50]
***Nelumbo nucifera***	n.d.	-	n.d.	-	-	-	-	-	-	-	-	Yang et al. [25]
*Olea europaea*	n.d.	-	n.d.	Q; W; D	n.d.	-	-	*Q (?)*	n.d.	n.d.	n.d.	Todd and Bretherick [50]
***Papaver rhoeas***	n.d.	-	n.d.	-	-	-	-	-	-	-	-	Yang et al. [25]
*Phoenix dactylifera*	n.d.	-	n.d.	-	n.d.	-	-	-	n.d.	n.d.	n.d.	Todd and Bretherick [50]
*Pinus contorta*	n.d.	Q; W; D	n.d.	Q; W; D	n.d.	-	-	*Q; W; D (?)*	n.d.	n.d.	n.d.	Todd and Bretherick [50]
*Pinus mugo*	Q; W; D	-	-	n.d.	n.d.	n.d.	n.d.	n.d.	n.d.	n.d.	n.d.	Nielsen et al. [49]
*Pinus radiata*	n.d.	-	n.d.	-	n.d.	-	-	*Q; W; D (?)*	n.d.	n.d.	n.d.	Todd and Bretherick [50]
*Pinus sabiniana*	n.d.	-	n.d.	-	n.d.	-	-	*Q; W; D (?)*	n.d.	n.d.	n.d.	Todd and Bretherick [50]
*Polyfloral*	n.d.	-	n.d.	Q; W; D	Q; W; D	-	-	-	Q; W	-	Q; W; D	Atanassova et al. [40]
*Polyfloral*	n.d.	n.d.	n.d.	n.d.	n.d.	n.d.	-	-	-	n.d.	n.d.	Formicki et al. [44]
***Polyfloral***	n.d.	n.d.	n.d.	-	Q	-	-	-	-	n.d.	n.d.	Fuenmayor et al. [36]
*Polyfloral*	n.d.	-	n.d.	W	-	-	-	-	-	-	-	Kędzia [35]
*Polyfloral*	n.d.	n.d.	n.d.	Q; W; D	Q; W; D	-	-	-	-	-	-	Kostic et al. [39]
*Polyfloral*	n.d.	n.d.	n.d.	W	n.d.	-	n.d.	-	n.d.	-	-	Kump et al. [46]
***Polyfloral***	n.d.	-	n.d.	-	Q; W; D	-	-	-	-	-	-	Morgano et al. [34]
*Polyfloral*	n.d.	n.d.	n.d.	W; D	-	-	-	-	-	-	-	Villanueva et al. [23]
*Polyfloral*	n.d.	-	n.d.	n.d.	n.d.	-	n.d.	-	-	-	-	Sattler et al. [47]
*Polyfloral*	n.d.	n.d.	n.d.	n.d.	n.d.	n.d.	n.d.	n.d.	-	-	-	Silva et al. [48]
*Polyfloral*	n.d.	n.d.	n.d.	W	-	-	-	-	-	-	-	Szczęsna [24]
*Polyfloral*	n.d.	-	n.d.	-	n.d.	-	-	*Q; W; D (?)*	n.d.	n.d.	n.d.	Todd and Bretherick [50]
*Prunus communis*	n.d.	-	n.d.	-	n.d.	-	-	*Q; W; D (?)*	n.d.	n.d.	n.d.	Todd and Bretherick [50]
***Prunus dulcis***	n.d.	-	-	-	Q; W; D	-	-	-	-	-	-	Somerville and Nicol [51]
*Prunus persica*	n.d.	-	n.d.	-	n.d.	-	-	*Q; W; D (?)*	n.d.	n.d.	n.d.	Todd and Bretherick [50]
*Quercus kelloggii*	n.d.	Q; W	n.d.	-	n.d.	-	-	*Q; W; D (?)*	n.d.	n.d.	n.d.	Todd and Bretherick [50]
***Rapistrum rugosum***	n.d.	-	-	-	Q; W; D	-	-	-	-	-	-	Somerville and Nicol [51]
*Rosa rugosa*	n.d.	-	n.d.	W	-	-	-	-	-	-	-	Yang et al. [25]
*Rubus ulmifolius*	n.d.	n.d.	n.d.	n.d.	n.d.	n.d.	n.d.	-	-	-	-	Paulo et al. [52]
*Salix nigra*	n.d.	-	n.d.	W	n.d.	-	-	*Q; W; D (?)*	n.d.	n.d.	n.d.	Todd and Bretherick [50]
*Salix sp*.	n.d.	n.d.	n.d.	Q; W; D	Q; W; D	-	-	-	-	-	-	Kostic et al. [39]
*Salix sp*.	n.d.	-	Q; W; D	Q; W; D	Q; W; D	-	-	-	-	-	Q; W; D	Somerville and Nicol [51]
*Salix sp*.	n.d.	n.d.	n.d.	-	n.d.	-	-	-	-	n.d.	n.d.	Stanciu et al. [37]
*Salix sp*.	n.d.	Q; W	n.d.	W	n.d.	-	-	*Q; W; D (?)*	n.d.	n.d.	n.d.	Todd and Bretherick [50]
***Schisandra chinensis***	n.d.	-	n.d.	-	-	-	-	-	-	-	-	Yang et al. [25]
*Silybum marianum*	n.d.	Q; W; D	n.d.	Q; W; D	n.d.	-	-	*Q; W; D (?)*	n.d.	n.d.	n.d.	Todd and Bretherick [50]
*Sisymbrium officinale*	n.d.	-	-	-	Q; W; D	-	-	-	-	-	Q; W; D	Somerville and Nicol [51]
*Taraxacum vulgare*	n.d.	Q; W; D	n.d.	Q; W; D	n.d.	-	-	*Q; W; D (?)*	n.d.	n.d.	n.d.	Todd and Bretherick [50]
*Tea tree*	n.d.	n.d.	n.d.	n.d.	n.d.	-	-	-	-	-	-	Zeng and Yan [53]
***Trifolium balansae***	n.d.	-	-	-	Q; W; D	-	-	-	-	-	-	Somerville and Nicol [51]
*Trifolium repens*	n.d.	-	n.d.	-	n.d.	-	-	-	n.d.	n.d.	n.d.	Todd and Bretherick [50]
*Trifolium sp*.	n.d.	-	n.d.	-	n.d.	-	-	-	n.d.	n.d.	n.d.	Todd and Bretherick [50]
*Typha latifolia*	n.d.	-	n.d.	-	n.d.	-	-	-	n.d.	n.d.	n.d.	Todd and Bretherick [50]
***Ulex europaeus***	n.d.	-	-	-	Q; W; D	-	-	-	-	-	-	Somerville and Nicol [51]
***Vicia faba***	n.d.	-	n.d.	-	-	-	-	-	-	-	-	Yang et al. [25]
*Zea mays*	n.d.	-	-	-	Q; W; D	-	-	-	-	-	Q; W; D	Kostic et al. [45]
*Zea mays*	n.d.	-	-	-	Q; W; D	-	-	Q	-	-	Q; W; D	Somerville and Nicol [51]
*Zea mays*	n.d.	Q; W	n.d.	-	n.d.	-	-	*Q; W; D (?)*	n.d.	n.d.	n.d.	Todd and Bretherick [50]
***Zea mays***	n.d.	-	n.d.	-	-	-	-	-	-	-	-	Yang et al. [25]
*Zea mays*	-	-	-	n.d.	n.d.	n.d.	n.d.	n.d.	n.d.	n.d.	n.d.	Nielsen et al. [49]

## Discussion

The concentrations of N and P in honeybees are among the highest reported in invertebrates (cf. [54,55]). This observation is contrary to the common generalization that herbivores contain low amounts of N and P but is consistent with the data available for other Hymenoptera species [56]. Unexpectedly, these two elements are not among the most limiting elements due to the favorable *C*:*N*:*P* stoichiometry of pollen, which differs from that of other plant tissues (cf. [57–60]). However, deficiencies of Na, S, Cu, P, K, N and Zn in some pollen species may impose constraints on bee growth and development, precluding the production of stoichiometrically balanced jelly (the direct larval food produced by nurse bees from pollen, nectar and water). We emphasize that the stoichiometric mismatch and limitations are experienced during the immature stage, in which an organism is growing and developing; however, they influence the adult stage [2]. Adult bodies are already built; therefore, their functionality is limited mainly by energy. Nevertheless, the ability to build the fully functional, well-developed body of an adult may be influenced by the availability of non-energetic nutritional elements during the juvenile stage. Therefore, this limiting juvenile period in the life cycle of any organism may highly influence (1) the essential traits of an adult, such as size, fertility, lifespan, condition, and immunity and (2) ecological relationships in ecosystems [2,3,61]. Thus, such constraints may negatively influence the life history traits and fitness-related characteristics of honeybee individuals (size at maturity, condition, lifespan, etc.) [2,62], thereby having possible negative implications for the whole bee colony in terms of brood size, the health of the colony, and overwintering success [63,64].

The negative effect on bee condition and health by a diet composed of particular pollen species may be interpreted as the presence of toxic substances in pollen (e.g. [13, 65]). However, other explanations should also be considered, including inadequate quality of the diet and stoichiometric mismatch. In this context, Jones and Flynn [6] have shown that the observed “toxic” effect imposed on an organism (specifically: copepods) by a particular diet (specifically: algae) may in fact be caused by low nutritional value, such as an inadequate *C*:*N* ratio, rather than by toxins occurring in the diet. Such a “toxic” effect may be caused by stoichiometric mismatch, and the effects of the monospecific “toxic” diet may be overcome simply by mixing the diet with supplementary food sources of higher nutritional quality [6]. Using unialgal versus mixed algal diets of different nutritional status (*C*:*N* ratio) fed to a copepod, the authors showed that “toxic” algae (i.e., diatoms) are not toxic per se but that single-diatom diets are inadequate [6]. Similarly, here, we showed that for all studied elements except Na, the strategy of collecting pollen from various species should eliminate nutritional constraints. Polyfloral bee-collected pollen pellets were stoichiometrically balanced and, when sorted into morphospecies of different taxonomical composition, tended to be stoichiometrically unbalanced and showed limiting effects on bee growth and development imposed by the scarcity of various elements. Therefore, we suggest that plants should not be evaluated as adequate sources of bee food based solely on the quantity of pollen produced (e.g., [66,67]; cf. [68,69]). Pollen stoichiometry (i.e., quality) should be considered during such evaluations. Moreover, the occurrence and strength of stoichiometric mismatches (i.e., limitations on growth and development) in honeybees depend on caste and sex.

Bees favor certain species during pollen collection [22,70–72], and these preferences might be related to the “nutritional quality” of pollen; however, this term is ambiguous and has never been defined clearly (cf. [11,22,73]). Thus, the following question arises: what factors related to bee nutrition underlie high-quality pollen? It was recently shown that the foraging strategies of bumblebees may be shaped by the ratios of macronutrients in pollen [20,74]. Considering “nutritional quality” in the framework of ecological stoichiometry suggests that the mixing of pollen that differs in multi-elemental composition permits a stoichiometric balancing of the diet, thus avoiding limiting stoichiometric mismatches that constrain honeybee development. Bonoan et al. [12] showed that honeybee workers forage for essential minerals that may be lacking in the available food sources; these minerals include (1) a set of elements foraged in autumn when pollen resources are generally diminished and (2) Na in the summer when pollen resources are generally abundant. Here, we propose a possible explanation for such behaviors in bees, which may prefer foods that are rich in specific nutrients, allowing for a stoichiometrically balanced diet for the reared larvae. It should be emphasized that ecological stoichiometry considers organic molecules to be mutable entities that are built of immutable chemical elements [2,3]. While some elements (e.g., Na and K) play important roles as ions in insect physiology, other elements compose organic structures; for instance, heavy metals are contained in metalloproteins, and large amounts of P are used in rRNA [2,75,76]). It should be remembered that stoichiometric ratios reflect the proportions of various organic biomolecules, including different proteins, lipids, sugars, amino acids, RNA, DNA, and others, that constitute the considered biomass [2]. Studies of the foraging behavior and physiology of bees have shown that these organisms are able to respond to the nutritional values of pollen and nectar, both of which are nutritional rewards for pollinators [77,78]. Nicholls and Hempel de Ibarra [78], in a review concerning bees foraging on pollen, concluded that bees may taste pollen and that multiple floral cues have the potential to influence pollen collection. Moreover, the deficiency or richness of some elements in plants tissues might be indicated by visual (petal size and color) and odor cues. For example, the reduced attractiveness of the flowers of S-deficient plants to bees has been suggested [79,80], and S was indicated in the present study as one of the most limiting elements. We showed here that the concentrations of potentially limiting elements in pollen vary among taxa, and it is likely that honeybee growth and development are co-limited by a set of elements (mainly Na, S, Cu, P and K, possibly N and Zn). If so, the diversity of plant species, which leads to diversity in the stoichiometry of available pollen, may drive the growth of pollen-eater populations; by contrast, a decline in the diversity of plant species may lead to decreased numbers and diversity of pollen eaters.

Our results showed that except for Na, honeybee workers may be able to obtain a stoichiometrically balanced polyfloral pollen mix by merging pollen species that individually would not ensure stoichiometric balance. It is possible that particular plant species may be more essential for producing a stoichiometrically adequate pollen mix, as is suggested by the analysis of literature data (Table 7). A simple enrichment of randomly chosen plant species in certain habitats may increase the diversity of pollinating insects [81], whereas the withdrawal of specific plants may lead to a decrease in wild bee species; this latter factor has been suggested as a key driver of wild bee decline in The Netherlands [82]. Therefore, it may be possible that two factors influence the larval growth and development of bees: (1) general taxonomical diversity of pollen and (2) the availability of particular plant species that specifically produce stoichiometrically balanced pollen, even if these species produce relatively low amounts of pollen compared with other available species. Moerman et al. [83] showed in bumblebees that a colony may develop better on a mixed pollen diet than on monofloral pollen due to the more suitable nutrient combination, but this effect depends on the specific taxonomic composition of pollen rather than pollen diversity per se. The authors noted that it is possible for a specific monofloral pollen species (common broom) to have suitable nutritive values that result in better colony development than with a specific difloral diet (mountain ash and heather) that contains unsuitable nutritive contents and thus results in worse colony development. That study suggests that the availability of particular pollen species may be more important than the high diversity of the flora for bee growth and development. Those authors concluded that the chemical composition of a specific pollen species is a more important factor than pollen diversity for bumblebee development. However, that study considered only three pollen species, and more exhaustive comparisons are needed to elucidate the importance of specific pollen species in the bees’ diets. The question remains open as to what the effect of a truly polyfloral (mix of more than 3 species) diet might be. The literature analysis presented here suggested that the pollen of more than 20 out of 85 investigated taxa may be stoichiometrically balanced (Table 7). Plants producing such pollen may be important for oligolectic wild bees that rely on a particular taxon. For other bees, these plants may represent important species that mitigate limitations resulting from stoichiometric mismatch during the flowering period. The storing of such pollen by social bees would also prevent stoichiometric mismatches resulting from seasonal changes in flowering plants. Indeed, Hülsmann et al. [84] showed that the most important factor determining bee abundance and diversity is plant species diversity, with specific taxa playing crucial roles. We hypothesize that the importance of plant species diversity for pollen eaters is indirect and may be associated directly with a diverse pollen stoichiometry. Based on our measurements and on data from the literature, we propose clover as a taxon producing pollen balanced for bees and therefore worth consideration in intervention strategies aimed at providing bees with adequate food base (see Figs 4–7 considering *PPM*2A, which is composed of approximately 99% *Trifolium* pollen, and Table 7 for the literature data). Moreover, plantations of single-species crops may weaken bee populations if these plants produce stoichiometrically unbalanced pollen. Honeybees tend to favor mass-flowering crops in agricultural habitats [85]. Studies of the stoichiometry of single-species pollen are needed to identify potential difficulties in the stoichiometric balancing of the diet when feeding on such pollen. Sunflower is a potentially limiting crop for the honeybee given the exceptionally low P concentration in its pollen (0.25–0.26%; [25,51]), and short longevity has been reported in bees that fed exclusively on sunflower pollen [86]. Todd et al. [66] identified sunflower crops as a resource useful for pollinators; however, the data presented by those authors only partially support this conclusion.

De Vere et al. [72] presented a list of 930 plant species belonging to 437 genera from which nectar and pollen were utilized by honeybees to varying degrees. This list provides extremely valuable data for use in choosing pollen species for further studies of the ecological stoichiometry of pollen eaters. Wood et al. [87], in reviewing the diets characterized in solitary bees on farmland, provided a list of plant taxa that may serve as pollen sources for oligolectic solitary bees. These sets of bee-plant taxa comprise good model systems for testing the hypotheses and ideas proposed in the present study. However, there are no data on within-species variation in pollen stoichiometry, and comparisons of within-species and between-species variations in pollen stoichiometry are needed, as environmental and human factors, especially soil quality and fertilization, might influence the pollen stoichiometry of a single plant species (stoichiometric homeostasis is weak in plants [2]).

Based on the mean concentrations of C in bee bodies and the potentially limiting elements (except Na, due to its low and insufficient concentration in pollen; see below) and the mean C concentrations in pollen pellets (as measured in this study), we estimated the minimal concentrations of these elements in pollen that would be required for stoichiometrically balanced pollen (i.e., that would give *TSR* values below 4; Table 8). Plant species producing pollen containing at least the concentrations of elements presented in Table 8 should promote the growth and development of bees. We chose N, P, S, K, Zn and Cu as potentially limiting elements because, using the original data presented here and the literature data, we found that the scarcity of these elements in pollen may limit bee growth and development.

**Table 8:** Estimated minimal concentrations (dry mass) of possibly limiting elements in stoichiometrically balanced pollen considering the body elemental compositions of honeybee queens, drones and workers.

Element	N	P	S	K	Zn	Cu
Minimal non-limiting concentration (d.m.)	3%	0.3%	0.2%	0.4%	26 ppm	7 ppm

The Na concentration in pollen is low and insufficient for bee development. However, Na concentrations in jelly reach values that meet or exceed the highest values reported in pollen pellets [88]. Even if 100% Na assimilation is considered, it is unlikely that bees concentrate the scarce Na available in pollen to a great extent, and nectar cannot serve as a source of nutritional elements other than C. Thus, it is likely that the bees supplement Na from sources other than pollen and nectar. For bees, the source of non-C elements may be “dirty water”, as honeybees are known to willingly utilize liquid waste and “dirty water”, which are rich in decomposing matter and salts [89,90]. We hypothesize that bees are able to produce stoichiometrically balanced jelly for the reared larvae by supplementing Na deficiencies with “dirty water”. Such supplementation has been suggested by Bonoan et al. [12]. Supplementation from the sweat, urine and excrement of animals is also possible [89] cf. [90].

Though stoichiometric mismatch should not be regarded as the single factor responsible for bee community declines, this factor should be taken into consideration. Certainly, specific organic nutrients are necessary for bee development. However, these mutable macronutrients are built of immutable elements. If food does not provide atoms of elements in the correct proportions, the well-developed body of an imago cannot be built.

By comparing the masses of elements that compose the bodies of various castes, we calculated the cost of production for each caste, which was expressed in elemental budget units. Drones are the “most expensive” to produce, as the highest masses of elements must be incorporated into their bodies (especially Cu, C, K and N), whereas individual workers are the “cheapest” to build. Considering the invested amounts of C, N, S and Mn, one worker is 3 times less expensive than a drone and 2.5 to 3 times less expensive than a queen. Considering K and Na, a worker is 2.7 and 2.5 times less expensive, respectively, than a drone and 2 and 3 times less expensive, respectively, than a queen. For other elements, the differences amount to 2–2.5-fold. A bee colony needs to produce large numbers of workers and drones during one breeding season [14,91] with a given amount of nutrients at its disposal. A portion of this amount must first be invested into a sufficient number of workers to allow the colony to survive [14,27]. The remaining amount of available nutrients may be invested in an unequal number of specimens belonging to another caste or sex. The queens are produced in such small numbers that the amount of this investment can be neglected. Thus, we hypothesize that the effective worker-to-drone ratio may depend on the stoichiometry of the available pollen. Considering the elements that are limiting to the development of all castes but particularly to drones (high *TSR* ratios, Figs 4–7), the ability to optimize the worker-to-drone ratio may be related mostly to the supply of Cu, S and K. Indeed, it was shown that drones are in general produced by bee colonies with good nutritional status [92].

It should be noted that the mass of the adult honeybee and its water content may be influenced by parasites (live wet weight tends to be higher but dry mass tend to be lower in non-infested bees vs. infested bees [93]). It has been reported that an ectoparasitic mite, *Varroa destructor*, may influence concentrations of proteins and carbohydrates in the bee bodies; however, the effect was weak and differed among various body compartments (R^2^ values ranged from 0.08 to 0.28; p<0.05 for head and abdomen, no significant effect for thorax [93]). It is not known what effect (if any) the bee infestation would have on the stoichiometry of the whole bee body, which is not affected by the live wet weight but only by the dry mass. In the present study, the level of parasitism was not investigated.

We presented data on bee-limiting elements in various species of pollen and indicated some plant species as highly valuable or highly undesirable for bees based on the framework of ecological stoichiometry. The approach used in our study relied on the biological model of *TSR*. Further direct experimental validation of the results presented in this study is needed.

## Conclusions

1. The feeding strategy of a pollen eater contrasts with the strategies of other herbivores due to the exceptional nutritional richness of pollen.
2. Particular honeybee castes and sexes differ in stoichiometry and need to incorporate various proportions of elements during development; therefore, different castes and sexes experience different stoichiometric mismatches and must be provided with appropriate and balanced combinations of nutrients in their food.
3. The nutritional elements that limit honeybee development to the highest degree due to their scarcity in pollen are Na, S, Cu, P and K. Zn and N were determined to be possibly limiting for bees.
4. Not all plants produce pollen that satisfies the nutritional requirements of bees with respect to the required proportions of elements. Pollen eaters may not be able to stoichiometrically balance their diets without access to pollen species that are rich in limiting elements. Floral diversity may allow all of the necessary nutritional elements to be gathered in the appropriate proportions. Thus, a diverse flora is needed for bee development.
5. Particular plant species that satisfy the nutritional requirements of bees may play greater roles than other species in stoichiometrically balancing the diets of pollen eaters. These taxa should be promoted in intervention strategies aimed at improving the nutritional base for pollen eaters, regardless of the amounts of nectar and pollen produced. We propose clover as such a stoichiometrically balanced taxon for bees.
6. Single-species crop plantations might limit bee development even if the crops are rich in nectar and pollen. Sunflower may negatively affect bees’ growth and development because of the scarcity of P in its pollen.
7. There is a need for data that allow the comparison of taxonomic, environmental, and soil nutritional status factors that may influence the elemental composition and stoichiometry of pollen.

## Supporting information

S1 Table Measured relative element contents in 3 honeybee castes/sexes (12 elements). (XLSX)

S2 Table Absolute element contents in 3 honeybee castes/sexes (12 elements). (XLSX)

S3 Table All of the pollen taxa composing the studied *PPMs*. % indicates the percentage of grains of a particular taxon in the total counted number of pollen grains in a particular *PPM*.
(XLSX)

S4 Table Measured relative element contents in 9 pollen pellet morphospecies (*PPMs*, 12 elements). (XLSX)

S5 Table Trophic stoichiometric ratios (*TSRx* = (*C*:*X*)Pollen/(*C*:*X*)Bee), where C = Carbon content and *X* = Content of element *x* in bees and the potential biomass source of larvae. Means, maxima, minima and percentiles were estimated using randomized resampling (N = 3000). *TSR* values above 4 denote limitations on development. Yellow indicates the values above the threshold value of *TSR* = 4.
(XLSX)

S6 Table Concentrations of elements in various hand-collected pollen and honey bee-collected pollen pellets, as reported in the literature. All the values are given in ppm d.m. Full citations are given in the second sheet.
(XLSX)
